# Direct Characterization of Thermal Nonequilibrium between Optical and Acoustic Phonons in Graphene Paper under Photon Excitation

**DOI:** 10.1002/advs.202004712

**Published:** 2021-05-01

**Authors:** Hamidreza Zobeiri, Nicholas Hunter, Ridong Wang, Tianyu Wang, Xinwei Wang

**Affiliations:** ^1^ Department of Mechanical Engineering Iowa State University Ames IA 50011 USA; ^2^ State Key Laboratory of Precision Measuring Technology and Instruments Tianjin University Tianjin 300072 P. R. China; ^3^ Institute of Chemistry Chinese Academy of Science Beijing 100190 P. R. China

**Keywords:** graphene paper, optical and acoustic phonons, phonon coupling factor, Raman thermometry, thermal nonequilibrium

## Abstract

Raman spectroscopy has been widely used to measure thermophysical properties of 2D materials. The local intense photon heating induces strong thermal nonequilibrium between optical and acoustic phonons. Both first principle calculations and recent indirect Raman measurements prove this phenomenon. To date, no direct measurement of the thermal nonequilibrium between optical and acoustic phonons has been reported. Here, this physical phenomenon is directly characterized for the first time through a novel approach combining both electrothermal and optothermal techniques. While the optical phonon temperature is determined from Raman wavenumber, the acoustic phonon temperature is precisely determined using high‐precision thermal conductivity and laser power absorption that are measured with negligible nonequilibrium among energy carriers. For graphene paper, the energy coupling factor between in‐plane optical and overall acoustic phonons is found at (1.59–3.10) × 10^15^ W m^−3^ K^−1^, agreeing well with the quantum mechanical modeling result of 4.1 × 10^15^ W m^−3^ K^−1^. Under ≈1 µm diameter laser heating, the optical phonon temperature rise is over 80% higher than that of the acoustic phonons. This observation points out the importance of subtracting optical–acoustic phonon thermal nonequilibrium in Raman‐based thermal characterization.

## Graphical abstract

The optical–acoustic phonon thermal nonequilibrium is first probed through novel electrothermal and optothermal techniques. For graphene paper, the optical‐acoustic phonon energy coupling factor is found at (1.59–3.10) × 10^15^ W m^−3^ K^−1^, agreeing well with theoretical predictions. Under ≈1 µm laser heating, the optical phonon temperature rise is over 80% higher than that of the acoustic phonons.

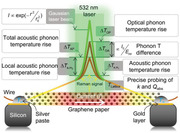

## 1 Introduction

2D materials have attracted significant attention due to their unique electrical, thermal, and optical properties.^[^ ^1^ ^]^ The applications of 2D materials tightly depend on the thermal behavior of 2D materials, especially thermal conductivity (*k*). Thermal conductivity influences device performance and lifetime significantly.^[^ ^2^ ^]^ Since the *k* of any given material relies mostly on its structure, characterizing it will also help us study the structure of the material, to be more specific, its phonons’ behavior. Raman spectroscopy is one of the main techniques used to characterize thermal transport inside 2D materials, and several Raman‐based methods have been developed to measure *k*.^[^ ^3^ ^]^ Most earlier works are based on steady‐state Raman (SS Raman). The SS Raman technique was introduced by the Balandin group to measure *k* of single‐layer graphene (SLG).^[^ ^4^ ^]^ In these works and other SS Raman related works,^[^ ^5^ ^]^ a two‐step experiment is conducted to track the temperature response of 2D materials: 1) measuring the Raman shift of a specific peak (*ω*) at different laser powers (*P*) to find *dω*/*dP*, and 2) a calibration that is necessary to relate the first step to the local temperature rise of the sample by finding *dω*/*dT*, where *T* is the sample's temperature. The thermal conductivity of several materials, such as graphene and various transition metal dichalcogenides (TMDs), is determined using this technique. However, calibration of Raman temperature dependence (step 2) and measurement of absorbed laser power introduce significant uncertainties in *k* measurements. In another Raman‐based technique known as time domain differential Raman (TD‐Raman), a square‐wave amplitude modulated laser is used with different heating periods and similar cooling periods. Then, a physical model determines temperature dependent Raman intensity, wavenumber, and Raman peak area. Finally, extracted Raman spectra are fitted using aforementioned relations to find the thermal diffusivity of a Si cantilever.^[^ ^6^ ^]^ In TD‐Raman, for very short heating times, e.g., less than 20 µs, performing the experiment is more challenging since it requires longer integration times to acquire a clear and resolved Raman signal. The frequency resolved Raman (FR‐Raman) technique overcomes this drawback by using pulsed laser heating, but in this case with similar heating and cooling periods.^[^ ^7^ ^]^ This makes the Raman signal stronger and more appropriate for peak fitting and the data analysis required for determining thermal diffusivity. Further development of Raman‐based methods was accomplished by introducing the energy transport state‐resolved Raman (ET‐Raman) technique.^[^ ^8^ ^]^ In the ET‐Raman technique, two different heating states were constructed: 1) steady‐state, using a CW laser, and 2) transient state, utilizing a picosecond, nanosecond, or CW modulated (FET‐Raman) pulsed laser. Under each heating state, the Raman shift power coefficient is measured as: *ψ* = *dω*/*dP*. Then, a new parameter called the normalized Raman shift power coefficient is introduced as Θ = *ψ*_transient_/*ψ*_steady‐state_. Depending on the sample structure (i.e., supported or suspended) and materials, and transient heating state used, *Θ* can carry information related to the thermal transport in the 2D material such as *k* of the 2D material, interfacial thermal resistance, and hot carriers’ effect. Note that *Θ* is independent of both the amount of laser energy absorbed and the Raman temperature coefficient, which makes this technique far more accurate than traditional steady state Raman techniques.^[^ ^8^, ^9^, ^10^, ^11^ ^]^

In all Raman‐based techniques reviewed above, a laser is used for both heating and exciting the Raman signal. During laser excitation, electrons inside the solid gain the photons’ energy and undergo an intense temperature rise. These hot electrons relax within a very short time, on the order of picosecond or less,^[^ ^12^ ^]^ and pass the majority of their excess energy to in‐plane phonons; therefore, they have a minimal contribution to thermal conduction.^[^ ^13^ ^]^ For instance, it is shown that the electron‐hole diffusion effect on thermal conduction inside a suspended WS_2_ nm‐thick film is less than 6% for a laser spot radius of ≈0.3 µm.^[^ ^11^ ^]^ Among the four different in‐plane phonon modes (LA, TA, LO, and TO), optical ones (LO and TO) receive most of the energy (more than ≈90%), and the remaining energy from the hot electrons is passed to in‐plane acoustic phonons through electron‐acoustic phonon scattering processes. LO and TO modes transfer their energy to the lattice via phonon–phonon (ph‐ph) scattering. In summary, after electrons get excited and relaxed, these are the optical branches that gain this energy and transfer it to acoustic phonons by scattering with other phonons. Through this laser heating process, the acoustic branches contribute most to heat conduction.^[^ ^14^ ^]^ Optical phonons have a negligible impact on conductivity due to their short lifetime, low specific heat, and low group velocity.^[^ ^15^ ^]^ Note that this does not mean these phonons are unimportant. Optical phonons play a significant role in the scattering of acoustic phonons, which leads to a decrease in thermal conductivity. Lu et al. determined the steady‐state and transient temperature profiles of different phonon branches of SLG and their energy coupling factor *G*_ph‐ph_ under laser irradiation using a multi‐temperature model (MTM).^[^ ^16^ ^]^ Their results showed significant nonequilibrium between phonon branches. This was attributed to the difference between electron‐phonon (e‐ph) and ph‐ph couplings strengths of these branches. Local nonequilibrium between optical and acoustic phonon branches are also reported by Sullivan et al. for suspended graphene layer.^[^ ^13^, ^17^ ^]^ Note that this thermal nonequilibrium is also observed in other nanomaterials, such as Si‐NCs, and is not only related to 2D materials.^[^ ^18^ ^]^

In a recent work by Wang et al., the temperature rise of phonon branches of MoS_2_, MoSe_2_, and graphene paper were probed and revealed that under steady‐state laser irradiation, the difference between the temperature rise of optical phonons and acoustic ones (Δ*T*_OP‐AP_) contributes more than 25% to the Raman‐probed temperature rise.^[^ ^19^ ^]^ Considering this contribution of Δ*T*_OP‐AP_, the intrinsic thermal conductivity of MoS_2_ and MoSe_2_ nm‐thick samples were determined by excluding the OP‐AP cascading energy transfer. Additionally, they characterized the *G*_pp_ (energy coupling factor) between optical phonons and acoustic phonons of MoS_2_ and MoSe_2_ in the order of 10^15^ and 10^14^ W m^−3^ K^−1^, respectively. *G*_pp_ of graphene paper were reported as ≈0.5 × 10^16^ W m^−3^ K^−1^.

In previous Raman‐based thermal conductivity measurement of 2D materials, especially graphene, the thermal transport inside the material was characterized based on the optical phonon behavior, while ignoring the nonequilibrium between optical and acoustic branches. These measurements show that the ZA mode contributes to the thermal conductivity of graphene significantly.^[^ ^13^ ^]^ Therefore, more studies are needed to consider this effect on the thermal characterization of 2D materials by focusing on the nonequilibrium between phonon branches, which imposes significant challenges in the energy transport study of these materials, as well as other nanomaterials and phase‐change materials, such as free‐standing nanowires.^[^ ^20^ ^]^

In this work, the nonequilibrium between in‐plane optical phonon modes (LO and TO) and acoustic modes of graphene paper (GP) is characterized and their local temperature difference under photon excitation is determined. Based on this, the energy coupling factor between these modes are determined. To perform this analysis, the intrinsic thermal conductivity (*k*), an essential parameter, is determined based on both electron temperature and optical phonon temperature with negligible interphonon branch nonequilibrium. It is shown that these two methods give close values, which confirms the accuracy of our measurement.

## 2 Results and Discussion

### 2.1 Physics on Distinguishing Optical and Acoustic Phonon Temperatures

**Figure 1:**
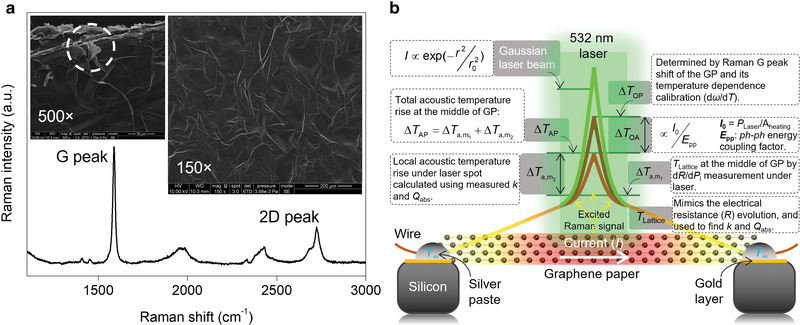
Materials structure and physical principles. a) Raman spectrum of the GP sample at room temperature. The very sharp G peak and lack of D peak indicate the high crystallinity of this GP. Insets show the SEM images of the top surface of the GP with 150× and 500× magnifications. The white dashed circle represents a GP flake detached from the bulk GP, showing that the forces between layers inside each flake are stronger than the forces between adjacent graphene flakes. b) The physical principles of the thermal nonequilibrium between optical and acoustic phonon branches of GP under laser irradiation, and schematic on how to distinguish their temperature rise and determine the energy coupling factor (*E*_pp_).

The GP sample used in this work was purchased from Graphene Supermarket and is composed of graphene flakes of 5–6 graphene atomic layers. More information about its structure and chemical composition can be found in Experimental Section. **Figure** **1**b shows the physical concepts of this work to illustrate the difference between optical and acoustic phonon temperatures. A GP sample is suspended over two electrodes, and its middle point is irradiated with a focused 532 nm wavelength laser beam (the green peak shown in the figure) which is also used for Raman signal excitation. The absorbed laser energy to excites electrons and brings them to an elevated temperature level. Then, electrons transfer their energy to optical phonons, which have very weak heat conduction. The majority of the absorbed laser energy is transferred from optical phonons to acoustic phonons. Acoustic phonons sustain the major macroscopic heat conduction and conduct the energy along the GP to the cold ends. As a result of this physical process, when radiation and convection to the surrounding air is negligible, the overall GP sample has a temperature rise and distribution: its temperature decreases linearly with the distance starting from the outside edge of the laser spot to both ends. The temperature rise at the middle point of the GP induced by the overall thermal resistance of the GP sample is designated as $\Delta T_{a,m_{1}}$. Because of the localized laser heating, the temperature within the laser spot heating region has another rise, designated as $\Delta T_{a,m_{2}}$. This temperature rise is the driving force for the heat conduction from the laser heating region to the surrounding nonheated regions. The overall temperature rise in the laser heating region will be $\Delta T_{\mathrm{AP}}=\Delta T_{a,m_{1}}+\Delta T_{a,m_{2}}$. These temperature rises are those of acoustic phonons and are controlled by the heat conduction and laser beam absorption. In the laser heating region, because of the cascading energy transfer from optical phonons to acoustic phonons, there is a temperature difference between them designated as Δ*T*_OA_. This one is determined by the local absorbed laser intensity and the optical‐acoustic phonon energy coupling factor *E*_pp_. Outside the laser heating region, there is no temperature difference between optical and acoustic phonons since no cascading energy transfer happens between them.

To distinguish the optical and acoustic phonon temperatures within the laser heating region, all the above‐mentioned temperature rises need to be precisely determined. The average lattice/acoustic phonon temperature rise (*T*_Lattice_) of the GP sample due to the heat conduction from the laser heating region to two ends is determined by measuring its electrical resistance change by passing a small electrical current through the sample. The local acoustic phonon temperature rise ($\Delta T_{a,m_{2}}$) in the laser heating region can be calculated using GP's known thermal conductivity (*k*) and the laser absorption *Q*_abs_ based on 3D high‐fidelity heat conduction modeling. The optical phonon temperature rise in the laser heating region (Δ*T*_OP_) is determined by measuring the Raman wavenumber shift (*ω*) and temperature dependence calibration (*dω*/*dT*) of G peak. With these determined temperature rises we are able to find the temperature difference between acoustic and optical phonons (Δ*T*_OA_), which is related to their energy coupling factor *E*_pp_.

To precisely determine $\Delta T_{a,m_{2}}$, which is a critical part in the work, the GP's thermal conductivity *k* and the laser energy absorption during laser heating needs to be determined with high precision. Two different methods based on the electrons' temperature rise and optical phonon temperature rise with negligible thermal nonequilibrium between optical and acoustic phonons are used to measure *k* of GP. The first technique relates the voltage evolution of GP during step‐current heating with its overall temperature evolution (without any laser heating), and therefore, its thermal conductivity. The second technique is able to determine *k* by relating the Raman shift (*ω*) evolution of the G peak under several electrical heating powers with the temperature rise of the GP at its center under laser irradiation. It is shown in the next sections that these two methods give very close results for thermal conductivity measurement. The amount of absorbed laser power (*Q*_abs_) is measured by studying the overall lattice temperature rise of the GP under laser heating and relating the laser power (*P*_l_) dependence of electrical resistance (*R*) with the temperature dependence of *R*. *Q*_abs_ is a very critical parameter in finding the acoustic phonon temperature rise under laser irradiation and needs to be determined precisely.

### 2.2 Intrinsic Thermal Conductivity of GP under Negligible Optical‐Acoustic Phonon Thermal Nonequilibrium

#### 2.2.1 Thermal Conductivity Determination Based on Electron Temperature

Two main parameters need to be evaluated with high accuracy in order to characterize the optical‐acoustic phonon thermal nonequilibrium: intrinsic thermal conductivity (*k*) and laser absorption (*Q*_abs_) of the GP sample. As will be shown, these two parameters play critical roles in the following experiments and the accuracy of our thermal characterization.

We first determine *k* of GP without introducing optical‐acoustic phonon thermal nonequilibrium. Such nonequilibrium is usually found in Raman‐based optical heating and sensing measurements. Here, *k* of GP is determined using two different techniques: transient electrothermal (TET) and steady‐state electro‐Raman‐thermal (SERT) techniques. The thermal conductivity (*k*) of GP has been characterized in detail in several works, but still could vary between different GP samples.^[^ ^21^, ^22^ ^]^ Xie et al. reported *k* of similar GP material in the range of 634–710 W m^−1^ K^−1^ for different samples.^[^ ^22^ ^]^ Therefore, it is necessary to measure the *k* of the GP used in this work for precise optical‐acoustic phonon nonequilibrium study.

**Figure 2:**
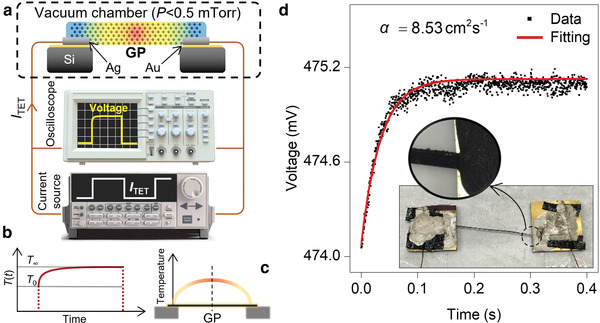
Thermal conductivity characterization of GP based on electron temperature. a) Schematic of the TET technique for thermal conductivity measurement with negligible thermal nonequilibrium among electrons and phonon branches. The suspended sample is placed in a vacuum chamber, and a step current is used to heat the sample. Using an oscilloscope, the voltage evolution of GP is collected. b) The GP's transient temperature rise during TET measurement takes the same trend as the voltage rise. c) The spatial temperature rise distribution of the suspended GP during TET. d) Measured voltage rise of the GP sample during the TET experiment. Black points and solid red line represent the experimental data and fitting result. The inset of this figure shows the optical image of the suspended GP sample. Each electrode is a silicon substrate that is coated with a ≈100 nm layer of gold. Sufficient silver paste is added to both ends of the sample as well as lead wires to guarantee excellent electrical conduction through the sample and minimize electrical contact resistance.

The thermal diffusivity of the GP sample at room temperature (RT) is measured using the TET technique as follows. **Figure** **2**a shows the schematic of TET. A GP ribbon is suspended over two electrodes, and a DC step current (*I*_TET_) is generated using a current source (Keithley 2611A). *I*_TET_ is passed through the sample and induces joule heating inside the GP. The evolution of the voltage of the GP is monitored using an oscilloscope (Tektronix MDO3052). When the on‐time period of *I*_TET_ is long enough, the voltage of the sample experiences a transient increase just after applying *I*_TET_. Then, at longer times, it reaches a steady‐state condition. The GP sample is placed inside a vacuum chamber (environment pressure of less than 0.5 mTorr). Under this pressure, heat convection through the environment is negligible. Therefore, only heat conduction and thermal radiation contribute to the thermal evolution of GP. It is proved that for a small temperature rise of the sample, the voltage evolution of the sample mimics the temperature rise during each pulsed current heating.^[^ ^23^ ^]^ In other words, the temperature of GP increases by applying *I*_TET_, and this leads to an increase in electrical resistance (*R*) of GP, subsequently. As a result, the voltage between the two ends of GP will be increased, too. During the heating process, the average normalized voltage rise (*V* ^*^) at any time (*t*) is defined as V*(*t*) = [*V*(*t*) − *V*_0_]/[*V*_∞_ − *V*_0_], where *V*_∞_ and *V*_0_ are steady‐state and initial voltage, respectively. The normalized temperature rise (*T* ^*^), defined as T*(*t*) = [*T*(*t*) − *T*_0_]/[*T*_∞_ − *T*_0_], is equal to *V* ^*^. Here, *T*_∞_ and *T*_0_ are steady‐state and initial temperatures, respectively. The transient temperature rise of the GP sample along with *T*_0_ and *T*_∞_ are shown in Figure 2b. *T* ^*^(*t*) could also be written as below^[^ ^23^ ^]^

(1) $$T^{*}\left(t\right)=\frac{48}{\pi^{4}}\sum_{n=1}^{\infty }\frac{1-{\left(-1\right)}^{n}}{n^{2}}\frac{1-\exp[-n^{2}\pi^{2}\alpha_{\mathrm{eff}}t/L^{2}]}{n^{2}}$$

where *α*_eff_ and *L* are effective thermal diffusivity and length of suspended GP, respectively. Therefore, *α*_eff_ could be found using Equation (1) and the measured voltage evolution (*V* ^*^). Note that *α*_eff_ is a combination of real thermal diffusivity (*α*_real_) and the effects of thermal radiation. *α*_real_ is determined by subtracting the radiation effects from *α*_eff_ as: $\alpha_{\mathrm{real}}=\alpha_{\mathrm{eff}}-8\varepsilon_{r}\sigma {\bar{T}}^{3}L^{2}/\rho c_{p}\pi^{2}D$. Here, *ε*_r_, *σ*, *ρc*_p_, and *D* are emissivity, Stefan‐Boltzmann constant, volumetric heat capacity, and thickness of the GP sample, respectively. Also, $\bar{T}$ is the average temperature of GP over each heating cycle. Figure 2c shows the schematic spatial temperature rise of the suspended GP during TET. Since both ends of GP are connected to electrodes, the temperature rise at these two locations is zero, and it is maximum at the middle point during the electrical heating. More details about the theory of the TET technique can be found in our previous works.^[^ ^10^, ^23^, ^24^ ^]^

The inset of Figure 2d shows the optical image of the suspended GP over two silicon electrodes. Each electrode is coated with a layer of gold with 100 nm thickness. The electrical connection of the GP sample with electrodes is guaranteed by applying silver paste at both ends of GP. The voltage evolution of GP when *I*_TET_ is 150 mA is shown in Figure 2d. This plot shows that under this heating current, the voltage rise of GP is ≈1 mV. Experimental data are fitted using Equation (1), and the fitting result is shown by the red curve. Finally, *α*_real_ of GP is determined as 8.53 ± 0.02 cm^2^s^−1^. Note that the effect of radiation on thermal diffusivity is less than 2%. The length (*L*), width (*w*), and thickness (*D*) of this sample are measured as 16.07 mm, 248.8 µm, and 30.0 µm, respectively. The density of GP is also measured as 1676.4 kgm^−3^. Therefore, we are able to calculate the volumetric heat capacity of GP using the measured *ρ* and specific heat of graphite from reference values: (1.19 ± 0.01) × 10^6^ J m^−3^ K^−1^.^[^ ^25^ ^]^ Using measured *α*_real_ and *ρc*_p_ of GP, the *k* of GP is found as *k*_TET_ = *α*_real_ *ρc_p_* = 1015.1 ± 13.1 W m^−1^ *K* ^−1^. Based on the applied *I*_TET_ and measured *k*_TET_, the average temperature rise (Δ*T*_TET_) of the suspended GP during TET is obtained: $\Delta T_{\mathrm{TET}}=I_{\mathrm{TET}}^{2}RL/12wDk_{\mathrm{TET}}≃12.6\;K$. This temperature rise is only a fraction of the initial temperature (*T*_0_). Therefore, we can assume that under this Joule heating process, the resistance change is proportional to the temperature rise.

As mentioned in the introduction, first, the energy transfers from electrons to optical phonons. Then, optical phonons couple with acoustic ones and pass the energy to them. In TET, the difference between electrons’ temperature and acoustic phonons’ temperature is negligible compared to the determined temperature rise, as reported previously.^[^ ^19^ ^]^ This can be shown by estimating the temperature difference of electrons and acoustic phonons as $T_{e}-T_{\mathrm{AP}}=I_{\mathrm{TET}}^{2}RV^{-1}\left[{\left(\sum_{s}G_{e-\mathrm{OP}}\right)}^{-1}+{\left(\sum_{s}G_{\mathrm{OP}-\mathrm{AP}}\right)}^{-1}\right]$, where *T*_e_ and *T*_AP_ are electron temperature and acoustic phonon temperature, respectively. Also, here *V* is the volume of the GP sample. *G*_e‐OP_ and *G*_OP‐AP_ are coupling factors between electrons and optical phonons and the coupling factor between optical phonons and acoustic phonons, respectively. This temperature difference during the TET is estimated to be around ≈3.13 × 10^−7^ K which is negligible compared to the ≈12.6 K temperature rise of the GP sample in TET. Note that in the estimation above, the subscript *s* represents the phonon branch, and the coupling factor for each phonon branch is used from reference values^[^ ^13^ ^]^ where $\sum_{s}G_{e-\mathrm{OP}}$is 0.33 × 10^16^ and $\sum_{s}G_{\mathrm{OP}-\mathrm{AP}}$ is 0.45 × 10^16^ W m^−3^ K^−1^_._

#### 2.2.2 Thermal Conductivity Determination Using Optical Phonon Temperature

**Figure 3:**
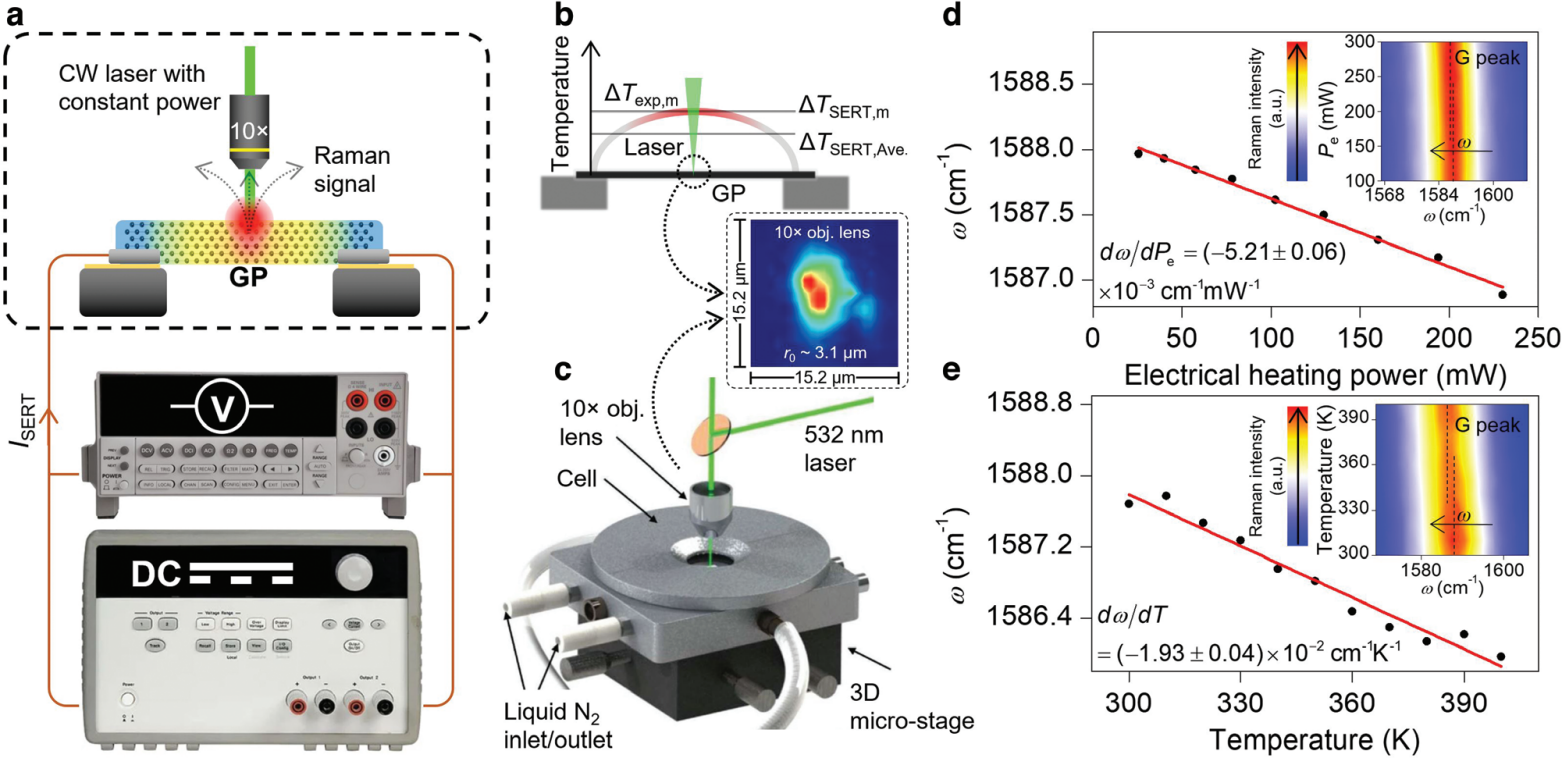
Thermal conductivity characterization of GP based on optical phonon temperature. a) Schematic of the SERT technique for direct thermal conductivity measurement with negligible thermal nonequilibrium between optical and acoustic phonon branches. The GP sample's middle point is irradiated with a CW laser (constant power) for Raman temperature measurement, and a DC current is passed through it with varying values to induce different electric heating powers (*P*_e_). The voltage over the two ends of GP is also read to find the actual *P*_e_. b) The average and middle point temperature rise of the GP during the SERT experiment. This figure's inset displays the spatial energy distribution of the laser beam under the 10× objective lens that is used for both Raman calibration and SERT characterization. c) Schematic of the environment cell chamber that is used to conduct the temperature dependence Raman calibration of GP. d) Result of SERT characterization to find *dω*/*dP*_e_. This plot shows sound linear relationship between *ω* of G peak and *P*_e_. The inset of this figure represents the 2D contour of Raman intensity of G peak and its redshift with increased electrical heating power. e) Result of Raman temperature calibration to find *dω*/*dT*. The inset of this figure displays the 2D contour of Raman intensity of G peak versus temperature, and the redshift of this mode with increased temperature. The uncertainty of Raman peak fitting is minimal and is less than 0.02 cm^−1^, therefore it is not shown in (d,e).

In this part, the thermal conductivity of the GP is determined directly by using the steady‐state electro‐Raman‐thermal (SERT) technique. This method is based on optical phonon temperature sensing whereas TET is based on the electron temperature.^[^ ^26^ ^]^ In this method, the middle point temperature of the suspended GP sample is measured based on both the temperature dependence and Joule heating power dependence of the Raman shift of G mode of GP. **Figure** **3**a shows the principals of this technique. A DC current is passed through the suspended GP (Agilent E3649A) to induce Joule heating while the center of the sample is irradiated using a continuous‐wave laser with low constant power. A constant irradiating laser power of 30 mW under the 10× objective lens is used to excite the Raman signal. Note that the absorbed laser power by GP is less than 30 mW. This process is conducted using several DC currents (*I*_SERT_), and Raman spectra of all electrical heating powers are collected using the Raman system. The voltage of GP (Keithley 2002) and it subsequent electrical resistance (*R*_SERT_) are recorded for each applied *I*_SERT_. Therefore, the electrical power (*P*_e_) dependence of wavenumber (*ω*) of G mode of GP is obtained as: *dω*/*dP*_e_. The steady‐state heat transfer equation under each *I*_SERT_ is

(2) $$k_{\mathrm{SERT}}\frac{\partial^{2}T\left(x\right)}{\partial x^{2}}+q_{0}=0$$

Here, *k*_SERT_ is the thermal conductivity of GP determined by SERT, and *q*_0_ is the heat generation per unit volume. *q*_0_ is found as $q_{0}=I_{\mathrm{SERT}}^{2}R_{\mathrm{SERT}}/V$. Using Equation (2), the temperature rise of the middle point (Δ*T*_SERT,m_) and average temperature rise (Δ*T*_SERT,Avg_) of GP are described as *q*_0_ *L*/8*k*_SERT_ *A*_c_ and *q*_0_ *L*/12*k*_SERT_ *A*_c_, respectively [Figure 3b]. *A*_c_ represents the cross‐sectional area of GP, as defined in the previous section (*wD*). Also, the temperature rise of the middle point of GP per heat generation (Δ*T*_SERT,m_/*q*_0_) is *L*/8*k*_SERT_ *A*_c_. The temperature of the middle point (Δ*T*_exp,m_) of GP per *P*_e_ is measured experimentally by Raman calibration of the temperature dependence of *ω*. To do so, the GP sample is placed in an environment cell chamber, and *ω* of the G peak of GP is collected over different temperatures for a constant laser power of 30 mW, as shown in Figure 3c. After this calibration, the Raman temperature coefficient *dω*/*dT* of GP is determined as (−1.93 ± 0.04) × 10^−2^ cm^−1^K^−1^. This value of *dω*/*dT* agrees very well with reference values.^[^ ^27^ ^]^ Figure 3e shows the result of this experiment and the linear fitting of *ω* versus GP's temperature in the range of 300–400 K. More information about the environment cell chamber and Raman system can be found in our previous works.^[^ ^28^ ^]^ The 2D contour of the Raman intensity versus temperature and Raman shift is indicated in the inset of this figure and shows the redshift of G peak with increased temperature.

**Figure 4:**
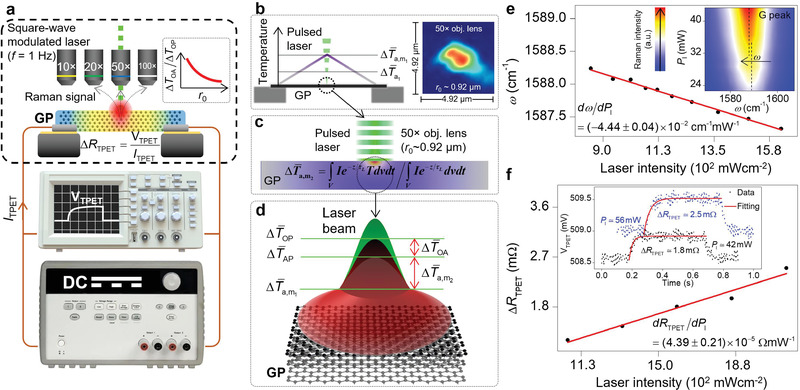
Thermal nonequilibrium between optical and acoustic phonons. a) Schematic of the TPET technique. While a constant 200 mA DC current is passed through the GP for transient resistance sensing, a square‐wave modulated pulsed laser under several objective lenses irradiates the center of GP for sample heating and exciting Raman signal. The voltage change under laser power *P*_l_ is monitored using an oscilloscope. The inset of this figure shows the fact that the contribution of Δ*T*_OA_ to total Δ*T*_OP_ decreases with increased laser spot size (*r*_0_). b) The lattice temperature rise of the GP under the pulsed laser. c) The lattice temperature rise contour of the center of GP under laser irradiation is calculated by our 3D numerical model. $\Delta {\bar{T}}_{a,m_{2}}$ is found based on this temperature contour using experimental values of *r*_0_. d) Schematic of different temperature rises discussed in this work under Gaussian laser beam heating. e) Result of Raman experiment in TPET measurement to find *dω*/*dP*_l_ of G peak of GP under the 50× objective lens. This experiment is conducted to find the temperature rise of optical phonons. The inset of this figure represents the 2D contour of Raman intensity of G peak under various irradiating laser powers (*P*_l_) and shows the redshift of this peak with increased *P*_l_. The Raman peak fitting uncertainty (Δ*ω*) is minimal and is less than 0.02 cm^−1^, therefore it is not shown in this plot. f) The laser power (*P*_l_) dependence of electrical resistance of GP (*R*_TPET_) measured in TPET experiment under the 50× objective lens. The inset of this figure indicates the transient voltage evolution of GP (*V*_TEPT_) when a constant 200 mA DC current is passed through it while its middle region is irradiated with a 1 Hz square‐wave pulsed laser under the 50× objective lens.

Figure 3d shows the result of the SERT experiment. Through linear fitting of *ω*‐*P*_e_ data, *dω*/*dP*_e_is found to be (−5.21 ± 0.06) × 10^−3^ cm^−1^ mW^−1^. The inset of this figure shows the 2D Raman intensity contour of the G peak for various *P*_e_ cases. This contour indicates that the G peak redshifts with increased electrical heating power while the Raman intensity remains the same due to the constant laser power of 30 mW over all *P*_e_ powers. Finally, Δ*T*_exp,m_ per unit *P*_e_ (mW) is found as Δ*T*_exp,m_/*P*_e_ = (*dω*/*dP*_e_)/(*dω*/*dT*) and is equal to 0.271 ± 0.006 K mW^−1^. Then, *k*_SERT_ is determined by equating Δ*T*_exp,m_/*P*_e_ to Δ*T*_SERT,m_/*q*_0_ resulting in 996.1 ± 21.60 W m^−1^ K^−1^. This value is very close to *k*_TET_ that was found in the previous section, and their difference is less than 2%. This firmly confirms the accuracy of both approaches to finding *k* of GP despite SERT measuring optical phonon temperature and TET measuring electron temperature. For the rest of this work, *k*_TET_ is used as the intrinsic thermal conductivity of GP in our analysis. The inset of Figure 3b represents the spatial energy distribution of the laser beam under a 10× objective lens that is used in both SERT and Raman calibration experiments. This energy distribution contour is analyzed by a Gaussian fitting method, and the effective laser spot radius (*r*_0_) at *e* ^−1^ of the peak value is determined as ≈3.1 µm. The spatial energy distribution under 10× and 50× [inset of **Figure** **4**b] are not perfectly circular, and this is caused by the rough surface of the GP sample. Based on the SERT results, the wavenumber dependence of electrical resistance (*dR*_SERT_/*dω*) is found as (−1.39 ± 0.02) × 10^−2^ Ω cm. Using this parameter and *dω*/*dT*, the temperature coefficient of electrical resistance is determined as $\frac{dR_{\mathrm{SERT}}}{dT=\left(\frac{dR_{\mathrm{SERT}}}{d\omega }\right)\times \left(\frac{d\omega }{dT}\right)}=\left(2.68\pm 0.01\right)\times 10^{-4}\Omega \;K^{-1}$. This parameter will be used in the following sections to find the laser power absorption coefficient.

It should be noted that SERT and TPET (next section) experiments are conducted in air and under atmospheric pressure. By estimating the effective thermal conductivity (*k*_conv_ *_+_*_rad_) that is induced by convection and radiation effects, it can be shown that it has a negligible contribution to thermal conductivity measurements. This is estimated as: $k_{\mathrm{conv}+\mathrm{rad}}=2L^{2}\left(4\varepsilon \sigma T_{\infty }^{3}+h\right)/\left(\pi^{2}D\right)$, where *ε*, *σ*, *T*_∞_, and *h* are emissivity, Stefan‐Boltzmann constant, room temperature (300 K), and natural convective heat transfer coefficient of air (≈5 Wm^−2^K^−1^), respectively.^[^ ^29^ ^]^ *k*_conv_ *_+_*_rad_ is estimated as ≈19.4 W m^−1^ K^−1^, which is less than 2% of the determined *k* from TET (or SERT), and shows that the effects of convection and radiation on our measurement are negligible.

In the previous paragraph, *k*_SERT_ of GP was determined based on the optical phonon (G peak) temperature by exciting the Raman signal of GP at several electrical heating powers. Under 30 mW laser irradiation, the nonequilibrium between optical and acoustic phonons is minimal, and we can assume it is negligible compared to the lumped acoustic phonon temperature rise. The temperature difference between optical and acoustic phonons under laser irradiation is estimated as $T_{\mathrm{OP}}-T_{\mathrm{AP}}=P_{\mathrm{abs}.l}V^{-1}{\left(\sum_{s}G_{\mathrm{OP}-\mathrm{AP}}\right)}^{-1}$, where *P*_abs.l_ is the laser power absorbed by GP. As will be shown in the next sections, the laser absorption coefficient for GP using a 10× obj. lens is ≈63%. When *P*_abs.l_ is ≈20 mW, the temperature difference between optical and acoustic phonons (*s*) is ≈3.7 × 10^−8^ K, while the average acoustic temperature rise over GP sample per unit *P*_e_ (in mW) is ≈0.18 K. And the average temperature rise of acoustic phonons over the GP sample varies in the range of 4.5–41.5 K during the SERT experiment which is much larger than Δ*T*_OP‐AP_. Note that similar phonon coupling factors from previous TET section are used here.

### 2.3 Strong Optical‐Acoustic Phonon Nonequilibrium under Intense Photon Excitation

In the following, a transient photo‐electrothermal (TPET) technique based on electrical thermal sensing and step laser heating is employed to determine the amount of laser absorbed and, subsequently, acoustic phonon temperature rise.^[^ ^30^ ^]^ Also, TPET makes it possible to distinguish the optical phonon temperature rise, and later, the difference between these two temperature rises that is necessary to determine the energy coupling factor between optical and acoustic phonon branches. Figure 4a shows the experimental setup and principles of TPET. The design of the experiment allows us to determine the exact amount of absorbed laser power, the optical phonon temperature rise, and the lattice temperature rise of the sample controlled by heat conduction.

In TPET, the GP sample is placed under several objective lenses, and its middle point is irradiated using a modulated CW laser with various laser heating powers. This is to vary the laser heating intensity which directly affects the optical‐acoustic phonon temperature difference without altering the heating of the GP sample. Here, the laser is modulated by a step function with 1 Hz frequency. The pulsed laser induces a transient temperature rise inside the sample which leads to a transient change of its electrical resistance (Δ*R*_TPET_). Simultaneously, a DC current (*I*_TPET_) is passed through the sample to sense this small electrical resistance change. The voltage evolution (*V*_TPET_) during each transient heating phase is observed using an oscilloscope. Therefore, Δ*R*_TPET_ at each laser power (*P*_l_) is obtained as $\Delta R_{\mathrm{TPET}}=\frac{\Delta V_{\mathrm{TPET}}}{I_{\mathrm{TPET}}}$. This experiment is conducted using several objective lenses (i.e., 10×, 20×, 50×, and 100×) to differentiate the effects of laser heating area. It will be shown that the temperature rise of GP and the amount of laser absorbed can be different under each objective lens. Finally, the laser power dependence of electrical resistance (*dR*_TPET_/*dP*_l_) is found. Additionally, Raman shift (*ω*) of G peak at each *P*_l_ and under all four objective lenses is collected to find the optical phonon temperature. Note that here *P*_l_ is the irradiated laser power just after the objective lens and it is not the absorbed power. The results of the 50× objective lens are used to demonstrate the analysis. The inset of Figure 4f shows the transient voltage change of GP during TPET for two different laser powers and the fitting data that are used to find the voltage rise during the transient laser heating. For all cases, a 200 mA DC current is used in order to observe this voltage rise. Figure 4f shows the laser power dependence of electrical resistance during TPET. This value is (4.39 ± 0.21) × 10^−5^ Ω mW^−1^ for the 50× lens. At this objective, the laser spot radius is ≈0.92 µm [Figure 4b]. The result of the Raman experiment is shown in Figure 4e, and *dω*/*dP*_l_ under the 50× lens is (−4.44 ± 0.04) × 10^−2^ cm^−1^ mW^−1^. The inset of this plot represents the 2D contour of Raman intensity versus *P*_l_ and *ω*, and shows the redshift of G peak when *P*_l_ is increased. Similar results for other objective lenses and the radius of the laser spot for each case are provided in **Table** **1**.

**Table 1:** The laser spot radii, irradiated laser power dependence of electrical resistance (*dR*_TPET_/*dP*_l_), and irradiated laser power dependence of Raman shift (*dω*/*dP*_l_) of G peak of GP in the TPET experiment

Objective lens	*r*_0_ [µm]	*dR*_TPET_/*dP*_l_ [× 10^−5^ Ω mW^−1^]	*dω*/*dP*_l_ [10^−2^ cm^−1^ mW^−1^)
10×	3.36	4.74 ± 0.05	−1.71 ± 0.03
20×	1.73	4.84 ± 0.17	−2.46 ± 0.03
50×	0.92	4.39 ± 0.21	−4.44 ± 0.04
100×	0.58	4.29 ± 0.17	−6.74 ± 0.08

This table shows that *dR*_TPET_/*dP*_l_ is almost identical under several objective lenses. *dR*_TPET_/*dP*_l_ depends on the lattice temperature rise of GP under laser irradiation, and as long as *P*_l_ is kept constant, the resistance change of GP will be intact, regardless of the objective lens that is used to perform the laser heating. Also, the increasing trend of the absolute value of *dω*/*dP*_l_ is due to the higher local temperature rise of the GP under the laser spot. In fact, *dω*/*dP*_l_ is proportional to the temperature rise of the optical phonons. This temperature rise is related to the laser heating area and is higher when the laser intensity is higher. It is relevant to smaller spot size of the beam on the sample and heat flux is higher for such situation.

#### 2.3.1 Optical Phonon Temperature

In this section, the optical phonon temperature rise ($\Delta {\bar{T}}_{\mathrm{OP},l}$) and the amount of laser absorbed (*Q*_abs_) in the TPET experiment are determined. $\Delta {\bar{T}}_{\mathrm{OP},l}$ in Kelvin per power of the irradiated laser (mW) is defined as $\Delta {\bar{T}}_{\mathrm{OP},l}=\left[\left(d\omega /dP_{l}\right)/\left(d\omega /dT\right)\right]$. Here, *dω*/*dP*_l_ and *dω*/*dT* were determined from TPET and Raman temperature dependence calibration, respectively. Under the 50× objective lens, $\Delta {\bar{T}}_{\mathrm{OP},l}$ is (2.30 ± 0.05) × 10^−2^ K mW^−1^. The optical phonon temperature rise of GP under other objective lenses are determined similarly and included in **Table** **2**. In the TPET experiment, the overall temperature distribution of the GP sample (from the middle to the ends) due to the pulsed laser heating that is monitored by the electrical resistance change (*dR*_TPET_/*dP*_l_) is linear, except in the area very close to the middle point, which is due to the local laser heating. This effect is discussed in the next section. Therefore, the average lattice temperature rise of GP ($\Delta {\bar{T}}_{a_{l}}$) is half of the lattice temperature rise under the laser spot (in the middle of GP) ($\Delta {\bar{T}}_{a,m_{1}}$) without the effect of local laser absorption. This is shown in Figure 4b. $\Delta {\bar{T}}_{a_{l}}$in Kelvin per power of the irradiated laser (in MW) under each laser objective lens could be found as $\Delta {\bar{T}}_{a_{l}}=\left(dR/dP_{l}\right)/\left(dR/dT\right)$, where *dR*/*dT* is the temperature dependence of electrical resistance as measured in the SERT experiment (= *dR*_SERT_/*dT*). Therefore, $\Delta {\bar{T}}_{a_{l}}$ and $\Delta {\bar{T}}_{a,m_{1}}$ of GP under the 50× objective lens are (0.16 ± 0.01) K mW^−1^ and (0.33 ± 0.02) K mW^−1^, respectively. $\Delta {\bar{T}}_{a_{l}}$ and $\Delta {\bar{T}}_{a,m_{1}}$ under other objective lenses are included in Table 2.

**Table 2:** Optical phonon temperature rise ($\Delta {\bar{T}}_{\mathrm{OP},l}$), average temperature rise of GP in whole domain ($\Delta {\bar{T}}_{a_{l}}$) and middle point ($\Delta {\bar{T}}_{a,m_{1}}$) per irradiated laser power, and laser absorption

Objective lens	$\Delta {\bar{T}}_{\mathrm{OP},l}$ [K mW^−1^]	$\Delta {\bar{T}}_{a_{l}}$ [10^−1^ K mW^−1^]	$\Delta {\bar{T}}_{a,m_{1}}$ [10^−1^ K mW^−1^]	*Q*_abs_ [%]
10×	0.89 ± 0.02	1.77 ± 0.02	3.54 ± 0.04	63
20×	1.27 ± 0.03	1.81 ± 0.06	3.62 ± 0.12	64
50×	2.30 ± 0.05	1.64 ± 0.08	3.36 ± 0.16	59
100×	3.49 ± 0.08	1.60 ± 0.06	3.20 ± 0.12	57

Additionally, *Q*_abs_ under laser heating in TPET can be written as $Q_{\mathrm{abs}.}=2\times \left[kA_{c}\left(\Delta {\bar{T}}_{a,m_{1}}/0.5L\right)\right]$, using the linear temperature distribution along the GP. Note that *Q*_abs_ is the laser power absorbed (in mW) per irradiated laser power (in mW). For the 50× objective lens, this value is 0.59. The *Q*_abs_ for other objective lenses are included in Table 2. These values are less than the theoretical values of the absorption rate of GP under a 532 nm laser by ≈25%. The absorption based on the refractive index of GP is defined as 1 − [(*n*_GP_ − *n*_air_)/(*n*_GP_ + *n*_air_)]^2^, where *n*_GP_ and *n*_air_ are refractive indices of GP and air, respectively. *n*_GP_ and *n*_air_ are ≈2.4 and ≈1.0, respectively.^[^ ^31^ ^]^ This shows that the theoretical value of the absorption is ≈80%, which is larger than the *Q*_abs_ found in this work. This is attributed to the structural differences between GP used in this work and references. Figure 1a also shows the SEM images of this sample and demonstrates that the structure of the sample is not perfect which can impact the laser light reflection and absorption.

As shown in Table 2, $\Delta {\bar{T}}_{\mathrm{OP},l}$ increases with decreased laser spot size, which is consistent with the *dω*/*dP*_l_ trend in Table 1 and the discussion that was presented there. Also, $\Delta {\bar{T}}_{a_{l}}$, $\Delta {\bar{T}}_{a,m_{1}}$, and *Q*_abs_% are very close for all laser heating areas. Any differences could be related to the experimental errors and uncertainties such as electrical resistance measurements at each *P*_l_. Based on the laser absorption, the optical phonon temperature rise ($\Delta {\bar{T}}_{\mathrm{OP}}$) in Kelvin per laser absorption (in mW) under 50× objective lens is (3.90 ± 0.08) × 10^−2^ K mW^−1^. The $\Delta {\bar{T}}_{\mathrm{OP}}$ under other objective lenses are represented in **Table** **3**.

**Table 3:** Raman weighted average temperature of optical ($\Delta {\bar{T}}_{\mathrm{OP}}$) and acoustic ($\Delta {\bar{T}}_{\mathrm{AP}}$) phonons and their differences ($\Delta {\bar{T}}_{\mathrm{OA}}$) in Kelvin per absorbed laser power by GP, as well as the energy coupling factor of in‐plane optical modes (*E*_pp_)

Objective lens	$\Delta {\bar{T}}_{\mathrm{OP}}$ [K mW^−1^]	$\Delta {\bar{T}}_{a,m_{2}}$ [K mW^−1^]	$\Delta {\bar{T}}_{\mathrm{AP}}$ [K mW^−1^]	$\Delta {\bar{T}}_{\mathrm{OA}}$ [K mW^−1^]	*E*_pp_ [10^15^ W m^−3^ K^−1^]
10×	1.41 ± 0.03	0.69	1.25 ± 0.01	0.16 ± 0.03	1.59 ± 0.30
20×	1.90 ± 0.05	1.23	1.79 ± 0.01	0.11 ± 0.05	8.76 ± 3.98
50×	3.90 ± 0.08	2.02	2.59 ± 0.02	1.31 ± 0.08	2.59 ± 0.16
100×	6.12 ± 0.14	2.80	3.36 ± 0.01	2.76 ± 0.14	3.10 ± 0.16

#### 2.3.2 Acoustic Phonon Temperature within the Laser Heating Region

The acoustic phonon temperature rise within the laser spot heating region consists of two parts: 1) overall temperature rise controlled by heat conduction ($\Delta {\bar{T}}_{a_{l}}$ and $\Delta {\bar{T}}_{a,m_{1}}$), and 2) the local temperature rise by laser absorption over the area under laser heating ($\Delta {\bar{T}}_{a,m_{2}}$). Therefore, the total acoustic temperature rise at the middle point ($\Delta {\bar{T}}_{\mathrm{AP}}$) is equal to $\Delta {\bar{T}}_{a,m_{1}}+\Delta {\bar{T}}_{a,m_{2}}$. From the previous section, $\Delta {\bar{T}}_{a,m_{1}}$ in Kelvin per absorbed laser power (in mW) is (0.57 ± 0.03) K mW^−1^ under the 50× objective lens. This was determined by measuring the electrical resistance change over the total length of the GP sample. Figure 4b shows the linear temperature rise distribution of suspended GP. Note that the heat conduction through the electrodes is much more than the GP; therefore, they are considered as the heat sinks without any temperature rise. The zoom‐in views of the center of GP under pulsed laser heating are represented in Figure 4c,d. Figure 4d illustrates the different components of the temperature rise in the middle of the sample. This graphic indicates the fact that just after laser irradiation the electrons transfer their excess energy to optical phonons, these phonons couple with acoustic branches, and acoustic phonon temperature is raised due to the laser absorption under the laser spot. Then, the heat is conducted by acoustic branches from the middle point to the heat sinks linearly. Therefore, the temperature rise in the middle of GP ($\Delta {\bar{T}}_{m}$) is equal to the optical phonon temperature rise ($\Delta {\bar{T}}_{\mathrm{OP}}$) measured using the TPET and Raman spectroscopy techniques. Note that $\Delta {\bar{T}}_{\mathrm{OP}}$ and $\Delta {\bar{T}}_{a,m_{2}}$ are average optical and middle point acoustic temperature rises under the Gaussian laser beam. Therefore, for each case, the $\Delta {\bar{T}}_{OA}$ obeys the Gaussian form of the laser beam, as shown in this figure.

So far, $\Delta {\bar{T}}_{a,m_{1}}$and $\Delta {\bar{T}}_{\mathrm{OP},l}$ are determined using SERT, TPET, and Raman spectroscopy techniques under different objective lenses (Table 2). A 3D numerical heat conduction model based on the finite volume method is conducted to find the second part of the acoustic temperature rise per absorbed laser power (in mW) under laser irradiation ($\Delta {\bar{T}}_{a,m_{2}}$). Note that $\Delta {\bar{T}}_{a,m_{2}}$ is the Raman intensity‐weighted temperature rise under laser excitation. Figure 4c shows the physics used to calculate $\Delta {\bar{T}}_{a,m_{2}}$ numerically. In this simulation, the temperature rise over the space domain is calculated under four objective lenses with the same laser spot radius (*r*_0_) as measured in the TPET experiment [Table 1]. Also, the laser absorption depth (*τ*_L_) of GP is calculated as *τ*_L_ = *λ*/4*πk*, where *λ* and *k* are laser wavelength and extinction coefficient of GP at *λ*, respectively. In this work, *λ* is 532 nm, and *k* is found from reference values as 0.817.^[^ ^32^ ^]^ Therefore, *τ*_L_ is 34.6 nm. *r*_0_ and *τ*_L_ are necessary parameters to find the Raman intensity distribution of the CW laser in space. Figure 4c shows the temperature contour of the GP sample under laser irradiation with the 50× (*r*_0_ ≃ 0.92 µm) objective lens that is obtained from numerical calculation. Note that this figure shows the cross‐sectional view of the GP at the middle point. Finally, the local temperature rise ($\Delta {\bar{T}}_{a,m_{2}}$) and total acoustic temperature rise under the heating area ($\Delta {\bar{T}}_{\mathrm{AP}}=\Delta {\bar{T}}_{a,m_{1}}+\Delta {\bar{T}}_{a,m_{2}}$) are calculated as 2.02 K mW^−1^ and (2.59 ± 0.02) K mW^−1^ (per absorbed laser power), respectively. A similar procedure is conducted for the other objective lenses, and the final results are reported in **Table** **3**.

As shown in Table 3, $\Delta {\bar{T}}_{\mathrm{AP}}$ and $\Delta {\bar{T}}_{\mathrm{OP}}$ increase with decreasing *r*_0_. This is due to the fact that these two temperature rises are proportional to $r_{0}^{-n}$ when total laser energy is kept constant. Here n is equal to two for optical‐acoustic phonon temperature difference. Note n is less than two for the acoustic temperature rise because it depends on the thermal conductivity of GP as well as laser spot size (*r*_0_), and the strong heat conduction of acoustic phonons weakens the effects of the heating area on $\Delta {\bar{T}}_{\mathrm{AP}}$. The effects of the heating area on phonon temperature rise and their coupling factor are discussed in the following section in more detail.

#### 2.3.3 Optical‐Acoustic Phonon Energy Coupling Factor

As mentioned in the previous section, the local temperature difference between optical and acoustic phonons is proportional to the absorbed laser energy at any specific point in the space domain [*I*(*r*, *z*)]. As a result, Δ*T*_OA_ at any location (*r*, *z*) under the laser heating area can be written as Δ*T*_OA_ = *δI*/*E*_pp_, where *δ* (0 < *δ* < 1) and *E*_pp_ are the portion of absorbed laser energy transferred from the optical phonons to acoustic phonons and their energy coupling factor, respectively. Note here Δ*T*_OA_ is the difference between the two phonon branches temperature rise at each point in the space domain and $\Delta {\bar{T}}_{\mathrm{OA}}$ is the Raman intensity weighted temperature difference. This fact that Δ*T*_OA_ is proportional to *I* is justified and presented in our previous work by calculating the different acoustic and optical phonon branches temperature rise as well as the electron temperature rise using a multi‐temperature model (MTM).^[^ ^19^ ^]^ The Raman intensity‐weighted temperature rise of the acoustic and optical branches ($\Delta {\bar{T}}_{\mathrm{AP}}$ and $\Delta {\bar{T}}_{\mathrm{OP}}$) were determined in previous sections, and $\Delta {\bar{T}}_{\mathrm{OA}}$ could be found from their subtraction. In the following, the relationship between $\Delta {\bar{T}}_{\mathrm{OA}}$ and *E*_pp_ for the G peak of GP is found. The CW laser intensity in the space domain is expressed as

(3) $$I_{\mathrm{CW}}=\left(I_{0}/\tau_{L}\right)\exp\left(-r^{2}/r_{0}^{2}\right)\exp\left(-z/\tau_{L}\right)$$

Here, $I_{0}=1\;\mathrm{mW}/\pi r_{0}^{2}$ is the absorbed laser power per unit heating area at its center for 1 mW total absorbed laser. Using this equation and the fact that Δ*T*_OA_ = *δI*/*E*_pp_, the average temperature rise difference between acoustic and optical phonons is written as

(4) $$\Delta {\bar{T}}_{\mathrm{OA}}=E_{\mathrm{pp}}^{-1}\left[\frac{\int \int \tau_{L}^{-1}I_{\mathrm{CW}}^{2}\exp\left(-z/\tau_{L}\right)dV}{\int \int I_{\mathrm{CW}}\exp\left(-z/\tau_{L}\right)dV}\right]=\frac{\delta I_{0}}{3\tau_{L}E_{\mathrm{pp}}}$$

where *dV* is the unit volume of GP. Note that the term exp (−*z*/*τ*_L_) in both numerator and denominator represents the Raman signal dissipation when it leaves the scattering location. In this work, we used the G peak of the GP paper to probe the optical phonons in the TPET experiment, and since this peak is related to in‐plane vibrations, *δ* takes 0.94. This is based on the assumption that the energy is transferred mainly to in‐plane optical phonon branches (LO and TO) uniformly, as mentioned in the introduction section, and is consistent with multi‐temperature modeling of graphene^[^ ^13^ ^]^. Therefore, the energy coupling factor (*E*_pp_) could be found as $E_{\mathrm{pp}}=0.94I_{0}/3\tau_{L}\Delta {\bar{T}}_{\mathrm{OA}}$. Note that *E*_pp_ is related to both in‐plane modes of LO and TO combined. Based on this calculation, $\Delta {\bar{T}}_{\mathrm{OA}}$ and *E*_pp_ under the 50× objective are 1.31 ± 0.08 K mW^−1^ and (2.59 ± 0.16) × 10^15^ W m^−3^ K^−1^, respectively. *E*_pp_ under all objective lenses are listed in Table 3.

The increasing trend of temperature rises with decreasing laser radius was discussed in previous sections. As shown in Table 3, the accuracy of *E*_pp_ measurement is improved at higher objective lenses and is lowest for 10× and 20× cases. This is because $\Delta {\bar{T}}_{\mathrm{OA}}$ is proportional to *I*, which is related to the radius of the laser spot. Therefore, under these two cases, *I* is much smaller compared with the 50× and 100× cases, and the difference between the optical phonon temperature rise and the acoustic one is smaller, too. As a result, it becomes more challenging to distinguish them under larger laser spots. This effect is shown in the inset of Figure 4a, where the decreasing trend of $\Delta {\bar{T}}_{\mathrm{OA}}/\Delta {\bar{T}}_{\mathrm{OP}}$ versus *r*_0_ is indicated by a red curve. In the case of 20×, it is shown that the *E*_pp_ is larger than the other three cases that are in the same range, which is due to the small $\Delta {\bar{T}}_{\mathrm{OA}}$ under this objective lens. This could be caused by experimental uncertainty of *dR*_TPET_/*dP*_l_, *r*_0_, and *dω*/*dP*_l_ which affect $\Delta {\bar{T}}_{\mathrm{OP}}$ and $\Delta {\bar{T}}_{\mathrm{AP}}$ values.

### 2.4 Discussion

The coupling factor (*E*_pp_) that is determined and represented in Table 3 reflects the coupling between LO and TO modes with all acoustic branches. The values determined in this work are in good agreement with the theoretical calculations by Ruan's group.^[^ ^13^, ^16^ ^]^ In their work, the coupling factor between each phonon mode and lattice was obtained by a MTM method. For LO and TO modes, *E*_pp_ of single layer graphene was reported as 2.7 × 10^15^ and 1.4 × 10^15^ W m^−3^ K^−1^, respectively. Therefore, the total energy coupling factor from in‐plane optical modes to acoustic modes is around 4.1 × 10^15^ W m^−3^ K^−1^. The reported values in Table 3 are in good agreement with the theoretical calculations. This is especially true for the 50× and 100× objective lenses where the uncertainty of *E*_pp_ is less than 14% and 7%, respectively. The difference between the *E*_pp_ reported in this work and Ruan's work is mostly related to our approximation that the *δ* factor in Equation (4) is equal to 0.94 for LO and TO modes combined. This approximation simplifies the problem, but at the same time, introduces uncertainty to our analysis, too. In Ruan's work, single‐layer graphene (SLG) is used to model the nonequilibrium between optical and acoustic phonons, while in this work, as mentioned in section 4, the GP consists of graphene flakes that each have ≈5–6 layers. This is another factor that contributes to the difference between the determined *E*_pp_ in their work and our results. In the work by Wang et al., the *E*_pp_ between in‐plane optical phonons and acoustic branches was reported as 5.5 × 10^15^ W m^−3^ K^−1^ which is in the same range as our results.^[^ ^19^ ^]^ In that work, a relationship between optical phonon temperature rise and acoustic temperature rise were derived analytically as a function of laser spot radius. Also, the optical phonon temperature rise under several laser spot radii was found using a CW laser. By calculating the acoustic phonon temperature rise numerically and fitting the experimental data with the analytical solution, they could find *E*_pp_. Therefore, the difference between their result and the one represented in Table 3 could be due to the fitting uncertainty of temperature rise versus laser spot radius in that work.

It should be noted that the absorbed laser energy by each phonon is not totally used to increase the temperature of that phonon branch and part of it is passed to other phonon branches through several coupling processes. These processes are discussed in a work by Lu et al. as^[^ ^16^ ^]^

(5) $$\begin{array}{c} C_{e}\frac{\partial T_{e}}{\partial t}=\nabla \left(\kappa_{e}\nabla T_{e}\right)-\sum G_{\mathrm{ep},i}\left(T_{e}-T_{p,i}\right)+I/\tau e^{-z/\tau },\\ C_{p,i}\frac{\partial T_{p,i}}{\partial t}=\nabla \left(\kappa_{p}\nabla T_{p}\right)+G_{\mathrm{ep},i}\left(T_{e}-T_{p,i}\right)+G_{\mathrm{pp},i}\left(T_{Lattice}-T_{p,i}\right) \end{array}$$

Here, *C* and *κ* are volumetric heat capacity and thermal conductivity of each energy carrier, respectively. Also, *τ* is the optical absorption depth. *i* is the phonon branch index, and *e* and *p* denote electron and phonon, respectively. *G*_ep,_ *_i_* and *G*_pp,_ *_i_* refer to the energy coupling factor between electrons and phonons, and between phonon branches. *G*_pp,_ *_i_* of each phonon branch is related to *C*_p,_ *_i_* and the relaxation time of that phonon branch (*t_i_*) as *G*_pp,*i*_ = *C*_p,*i*_/*t_i_*. The relaxation time of optical and acoustic phonon branches of graphite and other carbon materials, such as carbon nanotubes, is in the order of ≈1–10 ps at room temperature.^[^ ^33^ ^]^ Therefore, the temperature rise of each phonon branch is related to the absorbed laser energy, as well as its coupling strength with other phonons, and cannot be written as only a function of its absorbed energy and volumetric heat capacity.

## 3 Conclusion

In summary, the thermal nonequilibrium between optical and acoustic phonons in suspended GP under laser irradiation was directly characterized. The intrinsic thermal conductivity of GP was determined using both electrons' temperature and optical phonons’ temperature while there was negligible thermal nonequilibrium among electrons, optical phonons, and acoustic phonons. Also, the laser absorption of the GP sample was measured precisely in order to characterize the acoustic phonon temperature rise. By combining several electrothermal and optothermal techniques, the optical and acoustic phonon temperature rises were determined, and the energy coupling factor between them was determined under different objective laser heating. It was shown that the thermal nonequilibrium between these phonon branches is more significant under smaller area laser heating. Under the 100 × objective laser heating, the optical phonon temperature rise was found to be over 80% higher than that of acoustic phonons. The energy coupling factor (*E*_pp_) between TO and LO optical phonons and acoustic phonons is found in the range of (1.59–3.10) × 10^15^ W m^−3^ K^−1^, agreeing well with the quantum mechanical modeling result of 4.1 × 10^15^ W m^−3^ K^−1^. Our results shed light on phonon–phonon interactions inside GP and their coupling strength and show that the nonequilibrium between phonon branches should be seriously considered in Raman thermometry techniques in order to uncover the intrinsic phonon energy transport.

## 4 Experimental Section

##### Structure Characterization of Graphene Paper

The GP used in this work was purchased from Graphene Supermarket and was used without any further modifications. Insets of Figure 1a show the SEM images of this GP with 150× and 500× magnifications. As indicated in these SEM images, the surface of the GP was not totally uniform and flat, and small ridges were visible. Also, the inset of Figure 1a on the left side shows that the forces between atomic layers inside each graphene flake were much stronger than the forces between stacked flakes. This is represented by a white dashed circle which shows that one layer on top of the GP is peeled off. Figure 1a displays the Raman spectrum of this sample at room temperature (RT). G and 2D Raman peaks were observed. The D peak was not observed in the Raman spectrum which demonstrates the high crystallinity of this sample. This Raman spectrum was obtained using a 532 nm CW laser (Excelsior‐532‐150‐CDRH Spectra‐Physics), and this laser is also used in the all experiments of this work. The GP was composed of graphene flakes. In the previous work, Raman spectra of this sample at 30 locations were obtained, and based on the ratio of the intensity of G peak to 2D peak (*I*_G_/*I*_2D_ ≃ 0.61 − 0.72), the number of graphene atomic layers inside each flake was found to be 5–6.^[^ ^22^ ^]^ Also, using X‐ray diffraction measurement, it was shown that the interlayer spacing of GP was 3.35 Å. This was equal to the interlayer distance of pristine natural graphite and shows that the GP has a highly ordered structure.^[^ ^34^ ^]^ As shown in the insets of Figure 1a, the GP sample was not totally flat. This surface roughness could affect the Raman measurement by affecting the laser spot size (i.e., laser heating area). However, as shown in Figures 3 and 4, laser spot radius was measured while each Raman data was collected. Therefore, the effects of surface inhomogeneity were minimized by precise measurement of laser spot size. In addition, this surface roughness will not affect the electrical resistance (*R*) measurement in TET and TPET, because in these methods, the overall response (change in *R*) of GP was measured and not just a specific flake. The chemical composition of this GP sample was characterized by X‐ray photoelectron spectroscopy (XPS) measurement as follows: C 1s (%98.91), O 1s (%0.66), and F 1s (%0.43). Results of XPS confirmed the high purity composition of GP and its highly carbonized structure.

## Conflict of Interest

The authors declare no conflict of interest.

## Data Availability

The data that support the findings of this study are available from the corresponding author upon reasonable request.
